# Understanding adherence to self-isolation in the first phase of the COVID-19 pandemic in England: a cross-sectional mixed-methods study

**DOI:** 10.1186/s12889-023-16674-9

**Published:** 2023-10-23

**Authors:** Charlotte Robin, Rosy Reynolds, Helen Lambert, Matthew Hickman, G. James Rubin, Louise E. Smith, Lucy Yardley, Shenghan Cai, Tingting Zhang, Piers Mook, Oliver McManus, Gemma Lasseter, Polly Compston, Sarah Denford, Juan Zhang, Richard Amlôt, Isabel Oliver

**Affiliations:** 1 Behavioural Science and Insights Unit, UK Health Security Agency, Liverpool, L3 1DS UK; 2 Population Health Sciences, Bristol Medical School, University of Bristol, Bristol, UK; 3 Department of Psychological Medicine, King’s College London, London, UK; 4 Psychology Department, University of Southampton, Southampton, UK; 5 UKHSA, Field Service, Health Protection Operations, London, UK; 6 Division of Health Sciences, Warwick Medical School, University of Warwick, Coventry, UK; 7 UKHSA, Field Service, Health Protection Operations, Cambridge, UK; 8 School of Psychological Science, University of Bristol, Bristol, UK; 9 Department of Anthropology and Archaeology, University of Bristol, Bristol, UK; 10 Behavioural Science and Insights Unit, UK Health Security Agency, Salisbury, UK; 11 UKHSA, Field Service, Health Protection Operations, Bristol, UK

**Keywords:** COVID-19, Self-isolation, Adherence, Behaviour

## Abstract

**Background:**

During the early “containment” phase of the COVID-19 response in England (January-March 2020), contact tracing was managed by Public Health England (PHE). Adherence to self-isolation during this phase and how people were making those decisions has not previously been determined. The aim of this study was to gain a better understanding of decisions around adherence to self-isolation during the first phase of the COVID-19 response in England.

**Methods:**

A mixed-methods cross sectional study was conducted, including an online survey and qualitative interviews. The overall pattern of adherence was described as never leaving home, leaving home for lower-contact reasons and leaving home for higher-contact reasons. Fisher’s exact test was used to test associations between adherence and potentially predictive binary factors. Factors showing evidence of association overall were then considered in relation to the three aspects of adherence individually. Qualitative data were analysed using inductive thematic analysis.

**Results:**

Of 250 respondents who were advised to self-isolate, 63% reported not leaving home at all during their isolation period, 20% reported leaving only for lower-contact activities (dog walking or exercise) and 16% reported leaving for higher-contact, and therefore higher-risk, reasons. Factors associated with adherence to never going out included: the belief that following isolation advice would save lives, experiencing COVID-19 symptoms, being advised to stay in their room, having help from outside and having regular contact by text message from PHE. Factors associated with non-adherence included being angry about the advice to isolate, being unable to get groceries delivered and concerns about losing touch with friends and family. Interviews highlighted that a sense of duty motivated people to adhere to isolation guidance and where people did leave their homes, these decisions were based on rational calculations of the risk of transmission – people would only leave their homes when they thought they were unlikely to come into contact with others.

**Conclusions:**

Understanding adherence to isolation and associated reasoning during the early stages of the pandemic is essential to pandemic preparedness for future emerging infectious disease outbreaks. Individuals make complex decisions around adherence by calibrating transmission risks, therefore treating adherence as binary should be avoided.

**Supplementary Information:**

The online version contains supplementary material available at 10.1186/s12889-023-16674-9.

## Introduction

In response to the global COVID-19 pandemic, governments around the world have placed great importance on contact tracing systems to minimise transmission by instructing those who had been exposed to self-isolate [1]. In the United Kingdom (UK) the isolation of people with symptoms and their contacts was vital in the early ‘containment’ phase of the pandemic response (January to March 2020), particularly before widespread testing was available. As a public health measure, isolation aims to prevent person-to-person spread of infections by separating people to interrupt transmission [2]. For COVID-19, this includes separating exposed from unexposed individuals, because of evidence of asymptomatic transmission [3, 4]. Adherence to these measures is essential to limit community transmission of the virus.

A growing body of evidence indicates variation in the extent to which people adhere to self-isolation guidance and what factors may influence adherence. Adherence to isolation in the UK has fluctuated over time, but appeared to be higher in the earlier phases of the pandemic. For example, self-reported complete adherence to strict isolation was around 25% in March 2020 [5, 6], but later surveys identified a decrease to around 18% by August 2020 [7]. Key factors associated with adherence include receiving social support during isolation [5, 6], regarding adherence to guidance as protecting the household and wider community [8, 9], having the ability to work from home [9, 10], confidence in government [11] and financial support [12].

However, understanding adherence to self-isolation is limited by how adherence is measured. There are no validated measures of adherence to self-isolation and it is generally measured as a self-reported binary outcome; adherent or not [5]. While measuring adherence in a binary way is useful for determining changes in adherence over time and providing rapid and pragmatic insights into behaviour, how individuals understand and adhere to self-isolation is likely to be more nuanced [9, 13, 14]. Reducing adherence to binary measures also misrepresents non-adherent behaviour as high risk, which is not necessarily accurate. It is also unclear how the public negotiate decisions around adherence to self-isolation guidance in the context of contact tracing, specifically when that advice has been provided directly to individuals by public health agencies.

During the first phase of England’s COVID-19 response (January to March 2020), contact tracing was managed by Public Health England (PHE), prior to the launch of the national NHS Test and Trace service in May 2020. Regional Health Protection Teams at PHE aimed to contact all known cases and their contacts to advise them of their status, provide them with information on self-isolation guidance and offer them support during their isolation period. Adherence to self-isolation during this phase and how people were making those decisions has not previously been determined. It is particularly important to understand adherence during this phase because it was characterised by so much uncertainty; the pandemic landscape was constantly shifting as a result of rapidly evolving knowledge about the virus. There is conflicting evidence on the role uncertainty has in adherence; uncertainty has been associated with lower adherence, possibly due to increased anxiety [15], confusion over symptoms [6], or unclear messaging [16]. However, uncertainty and fear were identified as a key communication challenge at the start of the pandemic [17] and worry about COVID-19 has also been associated with increased adherence [5, 7].

The aim of this study was to gain a better understanding of adherence to self-isolation advice in cases and contacts who were identified through contact tracing in England during the first phase of the pandemic response, when anxiety levels in the general population were higher than normal [18, 19]. Understanding factors affecting adherence during these initial phases of the national public health response is particularly important as high adherence to isolation gives the best chance of containing the virus before community transmission becomes widespread, and future emerging infectious disease outbreaks will be characterised by similar high uncertainty and high caution.

## Methods

This was a cross-sectional mixed-methods study of cases and contacts who were contacted by PHE’s Health Protection Teams in England in early 2020. All participants were sampled using the details recorded on PHE’s case management system (HPZone) and invited to take part in an online survey and follow-up qualitative interview. We undertook a mixed methods approach including a survey to maximise potential respondents to increase representativeness and generalisability of the findings. It was also essential to understand decisions around adherence in more detail therefore chose to complement the survey with qualitative in-depth interviews, which are better suited to an exploration of participant experience and perceptions.

### Case definitions for survey inclusion

Confirmed cases had a positive PCR test for SARS-CoV-2. Possible cases had a history of exposure (to a confirmed case or by reason of travel history) and symptoms of fever or dry cough or breathing difficulty. Contacts were people exposed to a confirmed case. For the purposes of our survey, we classified individuals based on the circumstances which would have prompted first contact from PHE.

### Sampling

All cases and contacts (as defined above) in England aged 18 years or over and entered onto PHE’s case management system ‘HPZone’ by 12^th^ March 2020 were potentially eligible. After applying exclusion criteria (Table S1 – Additional file 1), a total of 3616 people – 350 confirmed cases, 1472 possible cases and 1794 contacts – were invited to participate in the survey. Sample size was limited by the response rate.

### Survey

An online survey (Additional file 2) was developed using Snap Survey v11 (Snap Surveys, Bristol, UK), including sections on sociodemographic and household characteristics, self-reported adherence to advice received and self-reported barriers and facilitators to following advice. The survey was piloted among 15 cases and 15 contacts, and minor changes to wording were made to improve clarity.

### Interviews

Semi-structured interviews were used to explore experiences of self-isolation in more depth. A topic guide (Additional file 3) with open-ended questions was used to ensure key areas were covered but was used flexibly to allow exploration of new themes as they arose. The topic guide included sections on experiences of self-isolation, adherence to guidance, seeking information, advice and support. Interviews took place by telephone or online, were audio recorded and transcribed *verbatim*.

### Recruitment

#### Survey

The survey was completed in two phases with invitations sent on 24^th^ July 2020 and 9^th^ October 2020, approximately 6–9 months after participants were identified and contacted by PHE as part of contact tracing activities. The first phase invited 463 cases (232 confirmed, 231 possible) and 451 contacts. Due to a low response rate from the first phase, the second phase invited all remaining eligible cases (118 confirmed, 1241 possible) and contacts (1343). Invitations were sent via SMS, including a link to an online participant information sheet and the survey. A follow-up reminder SMS message was sent after 3–4 weeks; if no response was received after a further week, the invitee was recorded as a non-responder and no further contact was made. The survey could be completed anonymously, but respondents who consented to participate in voluntary follow-up qualitative interviews were asked to provide their contact details.

#### Interviews

Respondents who consented to interview were randomly selected to take part, stratified by status (case or contact) and index of multiple deprivation (IMD) quintile, based on home postcode. Overall, 78 respondents consented to interview, of whom 30 were invited and 16 interviews took place (all those who responded to the invite), between July and November 2020.

### Analysis

#### Survey

Analysis used Stata v15.1 (Stata Statistical Software 2017; StataCorp LLC, College Station, TX). Categorical data were described by percentage. Age was described by median and interquartile range; other continuous data were described by mean or categorised.

Variables taken from the case management system were age, status as case or contact, and quintile of index of multiple deprivation (IMD, a standardised measure) for the local area of their postcode. All other potential predictor variables were derived from responses to the survey (Additional file 2) and are listed in Table S3, Additional file 1. Most questions were developed specifically to apply to the novel circumstances of self-isolation in the UK in the first months of a coronavirus pandemic; they were piloted but not formally validated. Table S3 and its legend record how variables were dichotomised for analysis.

Respondents reported how often they left home for various reasons (Table 2) and responses were dichotomised as ever *versus* never for analysis. We categorised reports of leaving home during the isolation period into lower- and higher-contact outings, defining exercise and dog-walking as lower-contact and all other reasons (listed in Table 2) as higher-contact.

We used two approaches to investigate 62 factors for possible associations with adherence, dichotomising those with more than two categories (Table S4). Initially, we described the overall pattern of adherence as a categorical variable with three levels – never leaving home, leaving only for lower-contact reasons, and leaving for higher-contact reasons – and tested its association with each of the binary factors individually by Fisher’s exact test. Secondly, for factors showing some evidence of association in the initial analysis, we considered three separate binary measures of adherence – never (vs ever) going out for any reason, ever (vs never) going out for lower-contact reasons, and ever (vs never) going out for higher-contact reasons. For each measure, we estimated the risk ratio for each factor individually. Then, to explore differential effects of factors on lower- and higher-contact reasons for leaving home, we used seemingly unrelated estimation to compare the estimated risk ratios.

We used a deliberately inclusive criterion (P ≤ 0.1) to identify factors of potential interest for triangulation with qualitative insights.

#### Interviews

Transcripts were coded using an open approach i.e. codes were not decided a priori. This process disassembled data into discrete parts to develop a list of codes. Memos on emerging ideas and possible relationships between codes were kept alongside initial codes and codes that represented similar concepts were assembled into conceptual categories. Coding was performed iteratively within and between transcripts, using the technique of constant comparative analysis. The constant comparison between data and analysis allowed the development of codes, categories and theories to be tested across transcripts [20] until a final coding framework was developed. Consensus on the coding framework was reached through discussion with the study team. This coding framework was independently applied to the 200 free-text comments from the survey; no additional codes were developed during this phase of the analysis. Qualitative interviews were conducted by a PHE employee. Participants were aware of this and so reflexivity was exercised by taking into account possible influences of the interviewer background on responses when conducting the analysis. Analysis was conducted in Nvivo 11 (QSR International, London, UK).

## Findings

The overall response rate for the survey was 9% (322/3616), including 52 confirmed cases, 91 possible cases and 179 contacts: confirmed cases, older age groups, women and less-deprived localities were over-represented (Table 1). Of these, 250 reported being advised to self-isolate (survey Q7, first two options) and are included in analysis of adherence. Characteristics of interview participants are shown in Table S2; the majority (14/16) were female.

**Table 1:** Characteristics of survey invitees and respondents

Characteristic		All invitees *N* = 3616	Respondents *N* = 322
Case/Contact: N (%)	Confirmed case	350 (9.7%)	52 (16.2%)
	Possible case	1472 (40.7%)	91 (28.3%)
	Contact	1794 (49.6%)	179 (55.6%)
Age: median (IQR)	Years	42 (29–54)	48 (35–58)
Gender	Female	1862 (51.5%)	200 (62.1%)
	Male	1667 (46.1%)	118 (36.7%)
	Missing/prefer not to say	87 (2.4%)	4 (1.2%)
Index of multiple deprivation (IMD) quintile of local area:^a^ N (%)	1 (most deprived)	410 (11.3%)	24 (7.5%)
	2	631 (17.5%)	44 (13.7%)
	3	744 (20.6%)	80 (24.8%)
	4	785 (21.7%)	75 (23.3%)
	5 (least deprived)	937 (25.9%)	89 (27.6%)
	Missing	109 (3.0%)	10 (3.1%)
Ethnic group: N (%)	White^b^	Not available	287 (89.1%)
	All other ethnic groups^c^	Not available	33 (10.2%)
	Missing/prefer not to say	.	2 (0.6%)

^a^IMD defined for the local area (lower layer super output area) of participant’s postcode

^b^White (British/Irish/Other)

^c^Asian, Black/Black British, Chinese, Mixed, Other

Overall, of the 250 survey respondents who had been advised to self-isolate, most reported adhering to the advice (Table 2); 158 (63%) reported not leaving home at all during their isolation period and 51 (20%) reported leaving only for lower-contact activities i.e. exercise or dog walking. Forty-one (16%) left home for higher-contact reasons: shopping, medical appointments, childcare or meeting family and friends. Five (2%) had occasional visitors to their homes.

**Table 2:** Adherence to staying at home

Left home for given reason and frequency: N (%)^a^					
Reason^b^	Missing	Not applicable or not at all	Occasionally	More than half the days	Nearly every day
Shop – essential	0	225 (90.0)	23 (9.2)	2 (0.8)	0
Shop – other	1 (0.4)	248 (99.2)	1 (0.4)	0	0
Exercise	0	187 (74.8)	27 (10.8)	10 (4.0)	26 (10.4)
Medical	0	233 (93.2)	17 (6.8)	0	0
Work	0	250 (100)	0	0	0
Childcare/school	0	247 (98.8)	3 (1.2)	0	0
Help someone else	0	250 (100)	0	0	0
Meet people	0	246 (98.4)	3 (1.2)	1 (0.4)	0
Walk dog	0	230 (92.0)	7 (2.8)	4 (1.6)	9 (3.6)

Two responses citing ‘Another reason’ have been recoded into the categories above: “To have a covid test” is shown as ‘Medical’ and “Only to take the rubbish out and collect items left on doorstep” is treated as not going out at all

^a^Excludes 72 respondents who did not report being advised to isolate and so were not asked about going out

^b^See copy of survey (Q26) in Additional file 2 for full wording

Evidence from the survey supported the classification of exercise and dog-walking as lower-contact, implying lower-risk, activities than leaving home for any other reasons (listed in Table 1). The 51 people who left home only for dog-walking or exercise reported less contact with other people away from their own homes, compared with the 41 who went out for other reasons: only 18% vs 46%, respectively, ever spent time with people indoors, keeping > 2 m away; 10 vs 27% had closer indoor contacts; and 14 vs 51% had to touch surfaces other people had touched. (The low levels of indoor contact suggest that exercise was largely outdoors.) We found evidence that some factors had different patterns of association with lower- and higher- contact outings, indicating that respondents distinguished between them (Tables 3 and S4). The coloured arrows in Table 3 represent beneficial (light green) or detrimental (dark purple) directions of association between predictors and adherence behaviours. Larger, thicker arrows represent associations with evidence at the *P*≤0.1 level. Arrows point upwards for associations with greater frequency of the behaviour concerned, and vice versa.

**Table 3:**
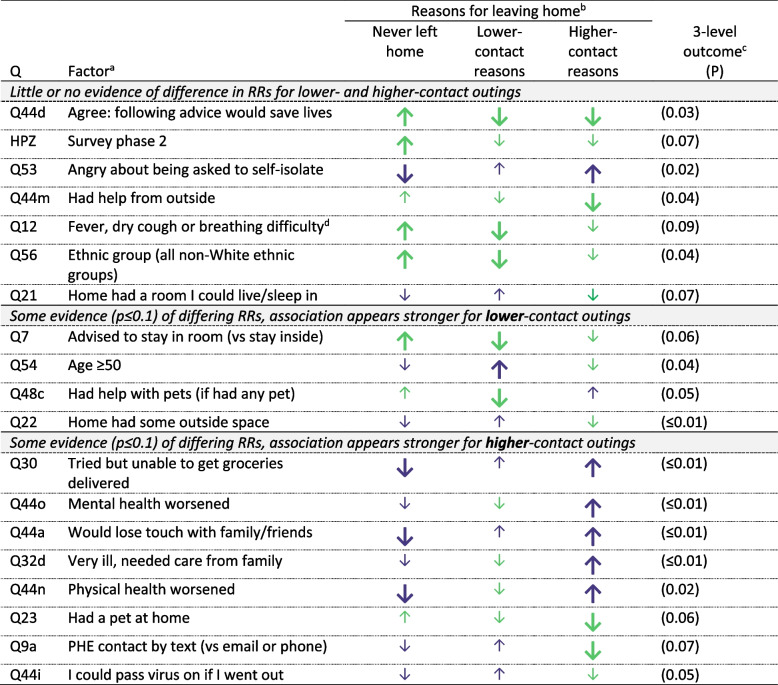
Factors showing some evidence (*p* ≤ 0.1) of association with leaving home for different reasons

*RR * Risk ratio. (See Additional file 1: Table S4 for all RRs, 95% CIs and *P*-values)

^a^The full wording of the relevant questions is in the survey, in Additional file 2

^b^The three columns under ‘reasons for leaving home’ relate to analyses for three separate binary outcomes: never (vs ever) leaving home; leaving home for lower-contact reasons (vs not); leaving home for higher-contact reasons (vs not). See Table S5 for risk ratios, confidence intervals and *P*-values

^c^The ‘three-level outcome’, the initial analysis, considered the pattern of respondents’ adherence to stay-at-home advice as a whole, classified as: never leaving home / leaving for lower-contact reasons only / leaving for higher-contact reasons (with or without lower-contact reasons as well).; *P* – Fisher’s exact test. See Table S4 for counts by category and predictor

^d^Symptoms recognised at the time as indicating Covid-19

Some evidence of association (*P* ≤ 0.1): a higher % of those with the factor report the behaviour (RR > 1)

Little/no evidence association (*P* > 0.1) but point estimate of RR > 1

Some evidence of association (*P* ≤ 0.1): a lower % of those with the factor report the behaviour (RR < 1)

Little/no evidence of association (*P* > 0.1) but point estimate of RR < 1

Arrow colours: light green = greater adherence (beneficial); dark purple = lower adherence (detrimental)

We found some evidence of association with the overall pattern of adherence – never going out, going out only for lower-contact reasons, going out for higher-contact reasons – for 19 dichotomised factors (Table 3). Those that relate to observations from the qualitative interviews are described in more detail below, alongside those insights. Evidence of association with going out for high-contact reasons was strongest (*P* ≤ 0.01) for perception of worsened physical or mental health or of loss of contact with family and friends, severe illness needing care from family, and inability to get groceries delivered; all these were linked to more frequent higher-contact outings. For lower-contact reasons, evidence of association was strongest for having been advised to stay in their room, non-White racial identity and agreeing that following advice would save lives; all these factors were linked to less frequent such outings. Evidence for *differential* association with going out for higher- and lower-contact reasons was strongest for having outside space at home, lack of grocery deliveries, severe illness, and worsened mental health.

### Shift in identity

During the early phase, all contact tracing was conducted by Health Protection Teams and therefore all participants were contacted directly by Public Health England (PHE) to inform them of their status as either a case or contact. Of 322 survey respondents, 43 (13%) recalled the reason for PHE contact being to inform them of a positive test result, 72 (22%) to ask about symptoms and arrange testing and 152 (47%) to inform of contact with a case (25 within their household and 127 outside). Of those who reported receiving advice, 204/250 were advised to “stay inside” and 46 to “stay in my room”.

The interviews revealed that receiving this contact resulted in participants experiencing a sudden shift in their identity, unexpectedly being classified as a case or a contact. This new identity brought with it certain rules and restrictions that they had to abide by. Their social world had abruptly become very different (Table 4, Quote 1).

**Table 4:** Experiences of contact tracing and self-isolation

Quote number	Code	Quote
Quote 1	Shift in identity	I’ve got a normally very active life and then suddenly everything stopped. I had nothing to look forward to. […] One minute you’ve got everything going on around you, I had lots of contact and social interaction, and then it’s just you. So it’s a very difficult thing, especially if you’re not expecting it either, is to get your whole head round the concept, isn’t it? Or how do you go round to suddenly doing nothing? (P11)
Quote 2	Symptom attribution	I think in some ways I felt so kind of surprised and kind of – a bit shell-shocked by it. I don’t know that I completely took it all in. (P15)
Quote 3		I was completely convinced I hadn’t got it, I just thought I was having a bad asthma, a bit of a cold because it was early March, it wasn’t the best weather. So I continued to sit with my family, because I share a house with my son, daughter in law and two grandchildren. Continued to mix with them, because I was completely convinced. (P12)
Quote 4		There’s no way I could have it, I’ve been really careful. I’ve had a brief conversation with her. She wasn’t stood right next to me, she was about a metre or so away. So in my mind, I had done absolutely everything I could possibly do to not catch it. (P12)
Quote 5	Conceptualisation of self-isolation: Guilt	I was at a party with everybody. I thought God, have I infected everybody? I think it's the guilty feeling. It's massively guilt-ridden feeling. You think who have I been with? Who have I already killed practically? And then when you go into lockdown, you're thinking, oh my God, am I going to kill my family, because they're the ones looking after me? And I can't do anything about it. You can't do anything about it, basically. It’s very bad in that way. (P4)
Quote 6	Conceptualisation of self-isolation: Sense of duty	I felt like I was doing my bit. I was following the rules, and it was absolutely 100% right and therefore do it. (P1)
Quote 7		You’re on your own. And you’re doing that to keep your family safe. (P7)
Quote 8	Renegotiating spaces in the home	On a daily basis there was inconveniences to negotiate and things you had to think about and navigate through. If I had to walk through the shared areas, to put gloves and a face mask on, which we made sure we adhered to. (P12)
Quote 9	Creation of boundaries	I never went out into their space or anything. We had a door between us for the whole time. (P4)
Quote 10		So I was concerned that when the post came in, for example, I was spraying the post and wiping the post. And when the food got delivered. I was worried I might just miss a bit and then we might get it anyway. (P5)

### Symptom attribution

For cases, the unexpected nature of the shift in their identity was related to how they conceptualised their symptoms. In some instances, despite knowing the case definition and having known exposure to a case or recent travel to a high-incidence country, there was still a sense of disbelief that the symptoms they were experiencing were actually COVID-19 (Table 4, Quote 2 and 3). One of these participants was also aware that a colleague had recently returned to work after visiting a high-incidence country, yet felt that the precautions she took in the workplace meant the symptoms she subsequently experienced could not be COVID-19 (Table 4, Quote 4).

This sense of uncertainty eased once cases were able to access testing. Of the 322 survey respondents, 96 reported definitely having had COVID-19, with 82 having this confirmed by a test. There was a high degree of trust in a remembered positive test result, 82/88 believing that they had definitely had coronavirus. However, there was stronger evidence of association with adherence to isolation guidance for symptoms than for a positive test, or report of having had COVID-19. The 95 respondents who remembered having fever, dry cough or breathing difficulty were less likely than others to have left home for lower-contact reasons (20% vs 33%), but there was no evidence of a difference in higher-contact outings.

### Conceptualisation of self-isolation

A part of the sudden shift in identity in becoming a case or contact was the realisation that they were now potentially a vector for the virus. In some cases, participants felt a sense of guilt over the potential risk of transmission and harm to others (Table 4, Quote 5).

#### Sense of duty

In addition to feeling guilty, participants accepted self-isolation as a way of mitigating risk of further transmission. They felt a sense of duty to protect others and this helped them to adhere to guidance (Table 3, Quote 6 and 7). This sense of duty was reflected in the survey, where 85% of respondents advised to self-isolate (212/250) agreed or strongly agreed that following the advice would help save lives. This belief was associated with greater adherence to self-isolation guidance and fewer reports of leaving home for any reason (33 vs 55%). Similarly, the majority of survey respondents (87%) agreed or strongly agreed that following advice to self-isolate would help to protect the NHS; however, there was no evidence that this belief was associated with adherence to the advice received.

#### Renegotiating spaces in the home

One way in which participants managed the risk they posed to people they lived with was through renegotiating spaces within their home. Following being identified as a case or contact, spaces within their home were subsequently designated clean or contaminated, which now had to be taken into consideration in their day-to-day lives, for example using personal protective equipment in shared spaces (Table 4, Quote 8). Similar behaviours were reflected in the survey respondents. Of 250 respondents advised to isolate, 76% reported washing their hands “nearly every time”, whereas only 48% of respondents reported cleaning surfaces and objects at the same frequency. Among people advised to stay inside (not in their room), such frequent handwashing was reported more often by people who lived with others (125/161, 78%), compared with those who lived alone (27/43, 63%).

#### Creation of boundaries

To help negotiate these contaminated spaces in their homes, participants created boundaries to reduce the risk of transmission, for example ensuring a barrier between the designated clean and contaminated spaces (Table 4, Quote 9). As well as keeping the virus within the confines of the home (or within certain spaces within the home), for some participants the boundary around the home had the dual purpose of keeping the virus out. For them, everything outside the home was potentially contaminated and they enacted a strict hygiene routine to try and minimise contamination (Table 4, Quote 10). Negotiating these competing boundaries highlights the complexity of everyday life in self-isolation.

### Maintaining a connection to the outside world

In addition to their sense of duty and a desire to protect others, participants also discussed several ways in which maintaining contact with the outside world could help them preserve the conceptual boundaries they had created and therefore help them adhere to self-isolation. Maintaining contact with the outside world was generally conceptualised in three ways; through social connectivity, tangible practical support and a sense of feeling known to public health authorities.

#### Social connectivity

Maintaining social connectivity was important for moderating the impact of self-isolation on mental health and wellbeing; this was either through socially distanced visits or virtually (Table 5, Quote 1). Participants also highlighted that maintaining a link with the outside world was particularly important as they were isolating early in the pandemic. As such, their experience was unique and support from others who were going through the same experience was important (Table 5, Quote 2). Similarly, survey respondents (37/250, 16%) who agreed or strongly agreed that following self-isolation advice completely would have caused them to lose touch with their friends or family were more likely to report leaving home for higher-contact reasons (35 vs 13%).

**Table 5:** Adherence to self-isolation

Quote number	Code	Quote
Quote 1	Social connectivity	Thankfully I had many visits from friends and family. They remained outside and we were able to have a chat at distance through the open top half of my stable door. These brief interactions definitely helped me through my period of self-isolation. (R1)
Quote 2		I think if somebody's going into isolation, it's really important that they have some people who can check that are going through the same thing. So, I would say if you can have WhatsApp groups set up where somebody can just join and say, this is how I'm feeling, because it really did help massively. Because I wasn't going through it alone at that point. (P4)
Quote 3	Practical support	We had some food delivered by friends whom we didn’t even answer the door to. They left it on the doorstep. Then after that we got an Asda delivery which again, that was a bit unknown because they were knocking at the door and we had to wave at the window to say to leave it and they didn’t understand. (R2)
Quote 4		I think it's support from the outside. If you are separated in your house, I mean, you may still get people now that have to separate in their houses. Having something outside is so important, but you do feel really guilty that you are going to give them the virus back. (P4)
Quote 5	Feeling known	I felt like I wasn’t forgotten. I felt like I was getting that daily contact. […] It kind of makes you feel a little bit special like oh you know, they’ve remembered me. The messages and the phone calls were reassuring because you knew that you weren’t forgotten about, but if there was something wrong, you’d be able to tell them. (P12)
Quote 6		Public Health has been really supportive and they’ve been interested, which I think it’s been fantastic to have that form of support. (P11)
Quote 7		I think having the texts coming in were incredibly helpful. Every day, I thought they were brilliant. To have it every day they to say, and your time is up. So, you do feel like… And also, I think that would help people stay in isolation. If you've got people who are just going, I don't care, I'm not going to be in isolation. If they feel like they're getting these texts, and they're being watched, it might make them stay in isolation. (P4)
Quote 8	Negotiating competing needs	The biggest problem I had at the very beginning was that I came straight back from a cruise, which was supposed to be 14 days and turned out to be 17 and then a two day journey back. I had no food in. I had nothing in my fridge. Yes I had a freezer and I had a food cupboard. (P11)
Quote 9		I didn’t really even think about getting shopping delivered really. Because of where I live it’s in the middle of nowhere and I’ve got like a student’s fridge, where you can fit about three meals in, so I needed to go shopping every day to just get the food for that day. So when this happened I didn’t really have anywhere to store anything, so I ended up living on Pot Noodles and soups, things that you could keep in the cupboard, which is a bit rubbish. (P9)

#### Practical support

The importance of maintaining a connection with the outside world was also highlighted for practical reasons, such as access to essential supplies including food and medication. At this stage in the pandemic, there were difficulties in accessing online grocery deliveries, as well as financial barriers due to minimum spend for deliveries at some supermarkets. Participants highlighted the importance of having a support network on the “outside” that could help with access to essentials (Table 5, Quote 3 and 4).

In the survey, grocery delivery was a clear facilitator of adherence. Delivery slots at this time were in short supply and 44 (18%) of 250 self-isolating respondents tried but were unable to secure one. They were distinctly more likely to have left home for higher-contact reasons than the 132 who did get deliveries and the 74 who did not try to (35% vs 10% and 16%, respectively) and – unsurprisingly – specifically for essential shopping (30% compared with 4% and 9%). More generally, 136/250 survey respondents (42%) agreed that they had received help from someone outside their home during their self-isolation period and, compared with others, they were less likely to leave home for higher-contact activities (11 vs 23%).

#### Feeling known

Feeling known to public health agencies also helped participants feel connected to the outside world during their isolation and this supported them to adhere to the guidance. Having the connection with someone in public health agencies, sometimes on a daily basis, helped reassure participants they had not been forgotten. This was particularly relevant for cases, who were sometimes anxious that they might need additional support if their symptoms worsened (Table 5, Quote 5 and 6).

In some instances, the regular contact from public health agencies resulted in people feeling that they were being monitored. Some participants suggested that the feeling of being known could help people adhere to self-isolation, even though they were not actually being checked (Table 5, Quote 7).

This was reflected in the survey where, after their first contact with the Health Protection Teams, 77 were contacted on some days and 107 every day during their isolation period (with the remaining 66 reporting no further contact). Of the 184 people who received further contact, 111 (60%) were contacted via text, 119 (65%) by phone and only 25 (14%) via email. Compared with the 73 who were contacted only by phone and/or email, there was some evidence that those whose further contact included text messages were less likely to leave home for higher-contact reasons (11% vs 21%). Text contact was more regular than phone contact, reported as ‘every day’ (rather than ‘some days’) by 79% (50/63) of those who had texts but not calls, 34% (24/71) of those receiving calls but not texts, and 69% of those who had both texts and calls. However, evidence of association with adherence was stronger for contact by text than for contact every day (Additional file 1, Table S2).

### Negotiating competing needs

Participants discussed needs which acted as barriers to being able to fully adhere to self-isolation, primarily the practical need to access and store sufficient food. The sudden shift from normal life to the constraints of being a case or contact meant they were unprepared for a two-week isolation period (Table 4, Quote 8). Some participants’ homes presented additional barriers such that they could not adequately prepare, even if they had known they needed to isolate – for example, not having a big enough fridge to store sufficient food (Table 5, Quote 9).

### Rational adaptations to mitigate risk

As a way of negotiating these competing needs, participants discussed making rational adaptations in their response to self-isolation guidance. These adaptations were focused on minimising risk of transmission of the virus, while still enabling participants to participate in the behaviours and routines they felt they needed to. For example, 16% of our survey respondents had no access to outside space at home, and some of them felt they needed to leave isolation so they could exercise outdoors, as this was important for their mental health. However, they purposely did this at specific times of day when they felt confident they would not come into contact with others (Table 6, Quote 1 and 2).

**Table 6:** Rational adaptations to mitigate risk

Quote number	Code	Quote
Quote 1	Rational adaptations to mitigate risk	Daily early morning walk or evening when no one is around helps to stay positive. Obviously this would depend on where you live but [town], as it is quite spread out, makes it easier to exercise outside while still staying away from others. (R3)
Quote 2		I think the only time that we left the house, other to go in our own garden. We went out once in the car. Just to escape the four walls. We stayed inside the car and just drove round the countryside for a short while and came back again. (P3)
Quote 3		Well, it’s the fact that he [the dog] wants to go out. To walk. If we didn’t stick totally to the go out for exercise once a day, then I would’ve found that difficult with him. I was going out 6:00 o’clock in the morning. Taking a good walk. Not seen a soul. And then taking him out around a bit later on and avoiding anybody you saw. If you saw anybody, it was the odd person. That was it. So, I must admit, I broke the rule with that. (P6)
Quote 4		I did, during my isolation, if I’m really honest, because we’ve got a dog, I would take him. I drove somewhere where I knew that I wouldn't see other people and took my dog for a walk. And did that, you know, because it was only fair to do that. But it was really trying to keep away from other people. (P2)
Quote 5	Over-adherence	It’s made me less inclined to go out. I’m definitely less inclined to go out. I think you develop a bit of a safety bubble, whether it’s consciously or not. And you just know that your home is your bubble. So it makes you less likely to want to expose yourself. It definitely makes you sub-consciously create your own space and not necessarily want anything to penetrate that. You want to stay very much where you know you’re safe. (P12)
Quote 6		I suppose a bit nervous. Not nervous, that’s not the right word, that kind of like apprehensive feeling. It doesn't take long to create habits, you know, like I suppose two weeks had felt like long enough for it to feel a bit overwhelming when we went outside. (P10)
Quote 7		I think in some ways it has made us kind of sealed off and reluctant to get back to some sort of normal. So we’re tending to keep our little sealed bubble going for now. (P12)

Similar competing needs were highlighted around pet ownership, specifically dogs. Overall, 43% (108/250) of survey respondents reported having a pet, primarily dogs (28%) and cats (22%). Those who had a pet at home were less likely to report leaving home for higher-contact reasons (10% vs 21%). Welfare concerns over their pets meant that in some cases participants did not fully adhere to self-isolation. However, their decision on *how* to break isolation guidance was based on minimising risk of contact with others, for example walking early in the morning or at places they knew would be quiet (Table 6, Quote 3 and 4).

#### Over-adherence

Some participants in both the survey and interviews reported over-adhering to the self-isolation guidance, during and after isolation. In the survey a quarter (40/161) of survey respondents who had received advice only to stay inside went beyond that and actually stayed in their room most days – 29 nearly every day, 11 on over half the days – and a further 25 (16%) did so occasionally.

In the interviews, some participants described how their experience of self-isolation had lasting impacts on their perceptions of COVID-19 risk and consequently their behaviour: they felt anxious following their self-isolation and were reluctant to leave the safety of their home (Table 6, Quote 5 and 6). In some cases, this resulted in over-adherence to COVID-19 guidance. For example, one participant discussed living with their “bubble”, which continued after their isolation period ended (Table 6, Quote 7).

## Discussion

Understanding how the public make decisions about following self-isolation guidance is important to ensure appropriate provision is in place to support adherence. Our study adds to the growing body of evidence that, despite frequently being reported simply as adherent or not, adherence to self-isolation and the decisions surrounding it are intricate and often in conflict with activities perceived as essential, such as buying food, exercising outdoors, and dog walking [9, 13].

The participants in our study understood the reasons for isolating – to reduce the number of contacts they had, and so reduce the risk of transmission. They then used this knowledge to make decisions around how to adhere to the guidance, based on balancing the perceived risk of transmission with maintaining their health and wellbeing (and that of companion animals) during isolation. This was facilitated by an assurance of care and connection, balanced with a sense of security provided by the state.

This indicates that people generally want to adhere to guidance and, while how they adhere may change over the course of the pandemic, intention will remain high. This has been reflected in other research, which has demonstrated that despite prolonged restrictions, engagement with personal protective behaviours continued throughout the pandemic response [21]. The nuanced decisions we identified people making at the start of the pandemic also demonstrates how voluntary behaviour change is effective; voluntary increase in frequency of hand hygiene behaviour has also been identified in the early stages of the pandemic [22]. Given the mounting evidence that enforcement can be ineffective at increasing adherence [23] and that voluntary measures have similar or improved adherence [24], supporting people to adhere to public health measures is an important consideration for pandemic preparedness policies. Our results suggest that in the UK, where enforcement of mandatory COVID-19 measures has been the final step in the policing strategy after engagement, explanation and encouragement have been attempted, a fifth 'e', for enable, should be added if people are to be supported to adhere to public health measures.

In our study, participants described the impact of their initial contact with public health authorities as resulting in a sudden shift in their identity to become a case or contact. This brought with it an acknowledgement they were now a potential risk to others and embedded within this was a sense of duty to protect those around them. For our participants, this sense of duty to protect others – primarily to save lives – acted as a motivator to adhere to self-isolation. While the majority of survey respondents also agreed that isolation would help protect the NHS, this did not have the same influence over adherence, suggesting participants did not necessarily associate protecting the NHS with saving lives. A sense of duty and desire to protect the community has been identified as a motivator to adhere to self-isolation previously [25] and is also a key principle in embedding behavioural science into public health campaigns, with emphasis on messages that promote mutual protection and collective solidarity [26].

The impact of being identified through contact tracing also highlights the importance of the knowledge and expertise of the public health teams doing the contact tracing – specifically being able to offer expert, professional support alongside isolation guidance. Participants in our study found that contact with public health teams helped them feel a connection with authority and gave them a sense of “feeling known”, which resulted in a feeling of security provided by the state; that those in authority cared about their wellbeing during isolation. This was also reflected in the survey, where regular text contact during isolation was associated with lower risk behaviour. This was particularly important during the early stages of the pandemic, when there was extensive uncertainty around the virus and the concept of self-isolation had not yet been embedded in the public consciousness. A sense of connection or “shared identity” with those in authority has previously been identified as a motivator for adherence to other health protective behaviours such as asymptomatic testing [27], as well as being an important factor in enhancing community resilience in response to emergencies [28].

Maintaining a connection with the outside world during isolation was also identified as a key motivator to adherence, specifically social connections and practical support. The way in which isolation was enacted by participants was to create a boundary around their living space (home or room within the home) to keep the virus within its confines. However, it is important to be able to maintain a connection with the world outside that boundary, without damaging its integrity. For the participants in our study, this included maintaining social connections, either virtually or socially distanced. Where participants felt they would lose touch with family or friends, they were more likely to leave home during isolation. Previous studies have identified how isolation resulted in psychological and emotional loss, highlighting the importance of maintaining social connections during isolation^14^. In addition, loneliness has been associated with disengagement with COVID-19 health protective behaviours previously [29], as people may prioritise leaving home to relieve feelings of loneliness resulting from adherence to isolation.

In addition to social support, practical support was also highlighted by our participants as a key facilitator for adherence to isolation, particularly access to essentials such as food and medicines. This emphasises the importance of providing tangible practical support for people who are isolating, including practical support from people outside the household [5], financial support [12] and access to mental health services and pet care services [30]. As the pandemic progressed, practical support became more available, both in the form of support from official organisations e.g. UK government offer of £500 financial support for those required to self-isolate [31] and via more informal mutual aid groups [32]. These groups often provided much of the local practical support not offered by official organisations, such as dog walking and as such became an essential part of the overall pandemic response.

Using binary measures for adherence has resulted in some studies reporting concerningly low self-reported adherence to self-isolation of 25.0% and 42.5% [5, 7], whereas other studies have reported much higher levels of 77.8% [33] and 90.0% [34]. In our study, strict adherence to isolation (not leaving the home at all) in people directly contacted by a public health team and instructed to isolate was 63%; however, when taking into account breaches to isolation that were perceived, correctly, as lower risk (dog walking, solo exercise outdoors), then adherence was over 80%. Reporting adherence as a binary outcome, without taking into account the complex decisions people are making about their isolation, may be problematic and fail to reflect real transmission risks. Nonetheless, there can be dangers involved in engaging in behaviours perceived as lower risk. In our sample, 10% of people who reported only engaging in lower-contact activities still reported being in contact with others indoors within a 2 m distance; a further 10% reported indoor contacts while maintaining at least 2 m distance. Lower risk is not no risk – and if self-isolation is to be used to quickly contain a future infectious disease outbreak, a focus on ensuring people better understand the risks of different activities may be required.

To understand these nuances in adherence, several studies have suggested alternative measures. For example, Fancourt et al. [13] differentiated between ‘complete’ adherence – those who *always* follow *all* the guidance – with ‘majority’ adherence – those who follow *some* of the guidance *some* of the time. In addition, Williams et al. [14] describe the difference between intentionally not following guidance (‘overt rule breaking’) and changing or interpreting guidance to suit individual circumstances (‘subjective rule interpretation’). Denford et al. [9] took this one stage further to explain the complexities of decision-making around adherence to social distancing and self-isolation and identified three patterns of adherence; caution-motivated super-adherence, risk-adapted partial adherence and necessity-driven partial-adherence. For those who partially adhered to guidance, Denford et al. found that these decisions were driven by two main factors. For some, decisions were based on personal perceptions of risk: behaviours considered to entail low risk of transmission (i.e. limited or no contact with others) were deemed safe and therefore partial adherence was justified. For others, decisions to break rules were based on tensions between an intention to adhere and a desire to stay safe on the one hand and a desire to maintain mental health or fulfil financial responsibilities on the other. In our study, there was little evidence that participants departed from guidance due to sheer disregard for rules, but rather they consciously and thoughtfully adapted it in order to carry out activities that they felt were essential or perceived as low risk.

Our study has contributed to an understanding of the intricacies of why and how people adhere to self-isolation advice. As such, incorporating public perceptions into the development of public health interventions is an important consideration for pandemic preparedness policies and using approaches such as the Agile Co-production and Evaluation framework [35], would ensure policies are effective and acceptable for the people they target.

## Limitations

Our study is based on a distinct sample of people, who were some of the first COVID-19 cases and their contacts in England, during the first phase of the pandemic response. While this enabled us to gather unique insights into experiences of self-isolation during the early stages of a global pandemic, the sample population is not representative of the wider population. During the early phases of the pandemic response, testing and contact tracing focused on returning holiday makers and travellers and their contacts; consequently, the sample population is primarily White British, of a similar age and from more affluent areas. We attempted to mitigate this by recruiting some interview participants from areas of lower IMD to ensure their experiences were included in the study and to improve generalisability of results. However, as the majority of our sample population were from more affluent areas, they may have experienced different barriers to self-isolation, compared with people for whom staying at home and accessing practical and social support may be more challenging.

The low response rate will have introduced response bias, and the delay between respondents being asked to isolate and inviting them to take part in the study (up to 6 months) will have resulted in recall bias as participants may not have been able to accurately recall their behaviours during the early stages of the pandemic. Recall may have been particularly challenging during the early stages of the pandemic, where there was so much uncertainty and rapidly changing advice and guidance; on the other hand, the experience of being identified as a case or contact for a novel virus and the associated identity shift that interview participants described clearly had the status of a memorable event for many. While this mixed methods study was able to provide some more detailed insights into the behaviours of people asked to self-isolate, our survey questions were not able to fully explore what contacts occurred and why, and it is possible that not all relevant activities and contacts were acknowledged and disclosed. It is also likely that participants who were more adherent were more likely to respond to the survey; concern about disclosing non-adherence or significant breaches of isolation may have discouraged less adherent people from participating in the study. However, many respondents in our study did disclose instances where isolation guidance was not fully adhered to.

For this analysis, we classified reasons for leaving the home pragmatically as higher or lower contact, as a proxy for potential risk of transmission. However, studies published since we designed the survey have refined our understanding of transmission risk; for example, risk from visiting a supermarket is lower than having people visit your home [36]. Future surveys should focus on the types of activities engaged in and places visited in more detail to relate adherence behaviour to transmission risk more accurately.

## Conclusions

The participants in our study demonstrated they were making rational adaptations in their response to self-isolation guidance, based on calibrating the risk of transmission and attempting to reduce contact with others as much as possible. Our findings highlighted that these decisions were driven by a sense of duty to protect others. Where isolation was not adhered to, breaches were often for reasons considered essential. The need for adequate practical, financial and social support during isolation has now been well documented; however, our findings highlight the additional impact of contact tracing on identity and feelings of ‘being known’ when asked to self-isolate. This emphasises that isolation cannot be viewed as a single intervention, but should be part of a complete test, trace and isolate process, where all components need to work together to support the desired outcome – reduction in transmission. A holistic system, where support is offered at the point of testing would improve adherence to isolation. Better understanding and support for nuanced decisions around adherence to isolation during the first phase of a national public health response to a pandemic, when uncertainty and anxiety is unavoidably high, is vital for pandemic preparedness for future emerging infectious diseases to ensure that early containment is effective.

### Supplementary Information

**Additional file 1:** **Table S1.** Exclusions. **Table S2.** Interviewee characteristics. **Table S3.** 62 potential predictors of adherence to self-isolation advice. **Table S4.** Factors ‘of interest’ showing some evidence (*p *≤0.1) of association with leaving home for different reasons.

**Additional file 2.** 

**Additional file 3.** 

## Data Availability

Data are available from UK Health Security Agency for researchers who meet the criteria for access to confidential data.
