# Supplementary material for: Understanding adherence to self-isolation in the first phase of the COVID-19 pandemic in England: a cross-sectional mixed-methods study
Source: BMC Public Health. 2023 Oct 23;23:2074. doi: 10.1186/s12889-023-16674-9 (PMC10594856; doi:10.1186/s12889-023-16674-9)
Supplement: Supplementary file 2 — Additional file 2. [file 12889_2023_16674_MOESM2_ESM.pdf]

Views on advice to self-isolate during the coronavirus (COVID-19) outbreak

Thank you very much for considering taking part in this study. Before you decide to take part, it is important that you understand why the research is being carried out and what it will involve. Please take the time to read the following information carefully and contact us if there is anything that is unclear, or if you would like more information.

Researchers from Public Health England, the University of Bristol and King's College London would like to understand more about your experiences of the coronavirus outbreak in England. Participation in this study will involve discussing your experiences of self-isolation and the impact this had on your mental health and wellbeing. The information you provide will help us provide support for people who are self-isolating.

You have been contacted because you were contacted by Public Health England as part of contact tracing activities. We are looking for male and female participants aged over 18 years to take part in the study. Participation in the study is completely voluntary and you are under no obligation to take part. We have invited you to complete an online survey. We are also looking for volunteers to take part in follow-up telephone interviews. If you would like to take part in an interview, please provide your contact details in the survey and a researcher will contact you to arrange a convenient time for you to take part in the interview. If you are selected to take part in an interview, you will be reimbursed for your time with a £20 shopping voucher.

For further details of the study, please review the information sheet:
https://snapsurvey.phe.org.uk/CoronavirusIsolationStudy/ ContactTracingGroupInfo.pdf

If you have any questions, please call 0151 706 6243 or email coronavirusevaluation@phe.gov.uk

Please note, you are under no obligation to take part and you are free to withdraw yourself and/or your data from the study at any time prior to data being analysed.

Consent statements

- Please read and consent to the following statements before starting the survey:
- I confirm that I have read and understand the study information above. I have had the opportunity to consider the information, ask questions and have had these answered satisfactorily.
- I understand that my participation is voluntary and that I am free to withdraw myself and/or my data from the study at any time prior to data analysis without giving any reason, and without my legal rights being affected.
- I understand that any information collected during this study (including identifiable information) will be held confidentially, in accordance with current data protection regulations.
- I understand that the outcomes of this study may be published in reports and journals, and that individuals participating in the study will not be identified in any of these reports.

We are also looking for volunteers to take part in follow-up telephone interviews about their experiences. If you would also like to volunteer to take part in a follow-up interview, please read and consent to the following statements and provide your contact details below or proceed to the survey.

- I understand that I will be asked to provide my name, phone number, and email address to allow the
- researchers to contact me for a follow-up interview and that this information will be deleted once the data has been analysed.
- I understand that during telephone interviews, audio recording will take place. I give permission for audio recordings to be taken of me during the interview. I understand that audio recordings will be used for evaluation purposes within this study alone, and that the audio recordings will be deleted once they have been transcribed.

Q1 First name:

Q2 Last name:

Q3 Telephone number:

Q4 Email address:

Q5 Overall, how worried are you about coronavirus? (Please select one option)

Extremely worried
Very worried
Somewhat worried
Not very worried
Not at all worried
Don't know

Contact with Public Health England

The following questions refer to when you were first contacted by Public Health England, between January and March 2020, to be informed that you had tested positive for coronavirus or come into close contact with a case, or to arrange testing, and the period you were advised to self-isolate for.

Q6 When you were first contacted by Public Health England, what reason were you provided with for being contacted? (Please select one option)

To inform me I had tested positive for coronavirus
To ask about my symptoms and organise testing
To inform me someone I was living with was a confirmed or possible case of coronavirus
To inform me someone I had come into contact with (outside of my home) was a confirmed or possible case of coronavirus
Other

Q7 When you were contacted by Public Health England, what did they advise you to do? (Please select one option)
Stay inside and avoid contact with other people for the isolation period
Stay in my room, only coming out when necessary (to use the bathroom or prepare food) for the isolation period
Do not self-isolate, but if symptoms develop then self-isolate and contact NHS 111
Other
If other, please provide details:

Q8 Following the first time you were contacted, were you contacted every day for the duration of your isolation period? (Please select one option)
Yes, everyday
No, I was only contacted some days
No, I was not contacted again

Q9 Following the first time you were contacted, how were you contacted during your isolation period? (Please select all that apply)
Text message
Email
Phone call

Q10 What did you think of the messages you received from Public Health England during your isolation period? (Please select one option)
Not at all useful
Slightly useful
Moderately useful
Very useful
Extremely useful

Q11 When I was contacted by Public Health England, I felt I received all the information I needed. (Please select one option)
Strongly agree
Agree
Neither agree or disagree
Disagree
Strongly disagree

Q12 During your isolation period, did you develop any of the following symptoms? (Please select all that apply)
A new continuous cough
A high temperature/fever
Shortness of breath/difficulties breathing
Runny or blocked nose
Aches and pains
Chest pains
Chills/shivering
Sore throat
Diarrhoea
Headache
Stomach ache
Feeling tired or having low energy
Loss of sense of smell or taste
None of these
Don't know

Q13 You reported a new continuous cough. Was this a: (Please select one option)
Dry cough
Wet cough (with mucus or phlegm)
Don't know

Q14 When your symptoms developed, what did you do? (Please select all that apply)
Waited a day or two to see if my symptoms got better, before making any changes
Checked what to do online
Called NHS 111
Called 999
Went in person to a doctor's surgery
Made a phone call to a doctor's surgery
Went to a walk-in centre
Went to a hospital's Accident and Emergency department
Went to a pharmacist/chemist
Stayed at home, not leaving home for any reason
Reduced the number of times I went out
Avoided contact with people inside my household
Avoided contact with people outside my household
Asked friends or family for advice
Did nothing
Don't know
Something else
If something else, please provide details:

Q15 Do you think you have had coronavirus? (Please select the option that BEST applies to you)
I have definitely had it
I think I have probably had it
I don't know if I have had it or not
I don't think I have had it
I have definitely not had it

Q16 Have you had a test for coronavirus? (Please select one option)
Yes, test said I had coronavirus
Yes, test said I didn't have coronavirus
No, never been tested

Household characteristics
Q17 Thinking back to when you were contacted by Public Health England, how many people were living in your household at the time (including you)? If you were living alone, please type "1".

Q18 At the time you were contacted by Public Health England, how many people in each age group did you live with (not including you)? Please enter 0 into any box for which there is nobody in your household in that age group.
Children aged 0-4 years
Children aged 5-17 years
Adults aged 18-69 years
Adults aged 70 years and over

Q19 At the time you were contacted by Public Health England, which of the following best describes your living situation? (Please select one option)
I was living by myself
I was living with family (this includes your partner, children or other family by birth or marriage)
I was living in shared accommodation
Other
Please provide details:

Q20 At the time you were contacted by Public Health England, did you share bathroom facilities at home?
Yes
No

Q21 At the time you were contacted by Public Health England, did you have a room in your home that you could live and sleep in without coming into contact with other people?
Yes
No

Q22 At the time you were contacted by Public Health England, did your home include access to any outside space, such as a garden, yard, balcony or terrace?
Yes
No

Q23 At the time you were contacted by Public Health England, did you have any pets that lived in your home?
Yes
No

Q24 Please provide details of pets that lived in your home: (Please select one option on each row) (Yes, No)
  Dog(s)
  Cat(s)

Bird(s)
Reptile(s)
Other

Experiences of self-isolation
The following questions are about your experiences during your isolation period, immediately following being contacted by Public Health England as part of contact tracing activities.
Please remember that your answers are always treated confidentially and are never analysed individually.

Q25 During your isolation period, did other people in your household stay at home and avoid contact with other people (i.e. self-isolate with you)? (Please select the option that BEST applies to you)
Yes
No
They tried, but were not able to
Not sure

Q26 During your isolation period, how often did you leave your home for each of the following reasons? (Please select one option for each row) (Not at all, Occasionally, More than half the days, Nearly every day, Not applicable)

- To go to the shops for groceries, toiletries or medicines
- To go to the shops for other items
- For exercise
- For a medical purpose excluding going to the shops/ pharmacy for medicine (e.g. an outpatient appointment)
- To go to work
- To take my child/children to and from school or daycare
- To help someone else (e.g. delivered medicine or done their shopping for them)
- To meet friends or family members who don't live with you
- To walk my dog
- For another reason

Q27 When you left home during your isolation period, how much time did you spend indoors with other people, but keeping 2 metres away from them? (Please select one option)
No time
Less than 15 minutes
Less than one hour
Several hours
One or more days

Q28 When you left your home during your isolation period, how much time did you spend indoors with other people and closer than 2 metres from them? (Please select one option)
No time
Less than 15 minutes
Less than one hour
Several hours
One or more days

Q29 When you left your home during your isolation period, how often did you have to touch any surfaces other people had touched (for example, to open doors or pay for things)? (Please select one option)
Never
A few times
Many times

Q30 During your isolation period, did you get groceries delivered? (Please select one option)
Yes
I tried, but was unable to access the website/book a delivery slot
I did not try to get groceries delivered

Q31 During your isolation period, did you have visitors in your home? (Please select one option)
Not at all
Occasionally
More than half the days
Nearly every day

Q32 During your isolation period, did any of these reasons make it difficult for you to self-isolate at home (this means staying on your own in your room with the door closed, only coming out when necessary (e.g. to use the bathroom or prepare food))? (Please select all that apply)
There was no room I could use to stay in on my own
I had to look after other people (e.g. children, old or sick family members)
Other family members wanted or needed to talk to or see me
I was very ill and so family members had to come in my room to look after me
None of these applied to me
Other

Q33 During your isolation period, how much of the time did you stay in your room with the door closed, only coming out when necessary (e.g. to use the bathroom or prepare food)? (Please select one option)
Nearly every day
More than half the days
Occasionally
Not at all

Q34 During your isolation period, did you use a separate bathroom or set up a bathroom rota?
Yes
No

Q35 During your isolation period, did you share hand towels or kitchen equipment with other people you were living with?
Yes
No

Q36 During your isolation period, was anyone in your household at higher risk from coronavirus because they were over 70 or had a health condition that could make them more seriously ill if they caught coronavirus?
Yes
No
Don't know

Q37 In some countries people with coronavirus, or people who are at higher risk of serious illness if they catch coronavirus, are offered accommodation to self-isolate outside their own home. This is done so that coronavirus does not spread to other household members, especially if they are at higher risk.

How likely would you have been to accept an offer of accommodation (for example, in an unused student flat) for you to self-isolate away from your home while you were ill? (Please select one option)
I definitely would have accepted
I probably would have accepted
I don't know if I would have accepted this offer or not
I probably would not have accepted
I definitely would not have accepted

Q38 At the time you were contacted by Public Health England, did you do any paid or voluntary work outside your home? (Please select all that apply)
I was self-employed, working outside my home
I was in full-time paid work outside my home
I was in part-time paid work outside my home
I was in full-time education and studying outside my home
I was in part-time education and studying outside my home
I was taking part in voluntary work outside my home
I was helping to care for someone outside my home (e.g. a friend or relative)
None of these applied to me

Q39 During your isolation period, did you continue to work outside your home?
Yes
No

Q40 Why did you continue to work outside your home? (Please select all that apply)
I worked as a key/critical worker
I could not afford to stop working
I was worried about losing my job
My employer asked me to go into work
I could not do my job from home
I had an important task that I needed to do in person (e.g. tend to animals, work in a lab etc.)
I wanted to reduce the workload of my colleagues
I needed to go into work to sustain my business
Other

Q41 During your isolation period, did you care for someone outside your home?
Yes
No

Q42 If you are advised to self-isolate for another two weeks, how likely do you think you would be to follow this advice? (Please select the option that BEST applies to you)
Highly unlikely
Unlikely
Neither unlikely or likely
Likely
Highly likely

Q43 How hard would it be to follow this advice? (Please select the option that BEST applies to you)
Very hard
Hard
Neither hard or easy
Easy
Very easy

Q44 When you were contacted by Public Health England, you were advised to stay at home and avoid contact with other people (self-isolate). Thinking about the advice to self-isolate, please tell us to what extent you agree or disagree with each of the following statements. (Please select one option for each statement) (Strongly agree to strongly disagree)
• If I had completely followed Public Health England's advice to self-isolate, I would have lost touch with my friends and family
• My friends or family would have disapproved if I had not completely followed Public Health England's advice to self-isolate
• If I didn't completely follow Public Health England's advice to self-isolate, I could have been in trouble with the police
• If I completely followed Public Health England's advice to self-isolate, it would have helped save lives
• If I completely followed Public Health England's advice to self-isolate, it would have helped protect the NHS
• If I had caught coronavirus, I may have become very ill
• If I had caught coronavirus, it would have had a severe impact on my family's wellbeing
• If I had completely followed Public Health England's advice to self-isolate there would have been more conflict with the people that I was living with
• If I had left home and met other people, I could have passed coronavirus to someone
• If I leave home and meet other people, I could catch coronavirus
• If I had completely followed Public Health England's advice to self-isolate it would have had a negative impact on how much money I had
• If I had completely followed Public Health England's advice to self-isolate I would not have been able to carry out important religious activities
• While I was self-isolating, I received help from someone outside my household
• Self-isolation made my physical health worse
• Self-isolation made my mental health worse
• Self-isolation made my physical health better
• Self-isolation made my mental health better
• I enjoyed spending more time at home during self-isolation

Q45 Thinking about people in England who were advised to self-isolate at the same time as you, who are around the same age as you, what percentage do you think fully followed Public Health England's advice? (Please enter a number between 0 and 100)

Q46 Thinking about people in the UK who are around the same age as you, what percentage do you think are fully following the Government's recommendations at the moment? (Please enter a number between 0 and 100)

Q47 During your isolation period, how often on average did you…

- wash your hands with soap and water, for more than 20 seconds
- cleaned objects or surfaces that you touched

(Options: More than half the times, Nearly every time, Occasionally, Not at all)

Q48 During your isolation period, did you...

- avoid contact with your pet(s)
- wash hands immediately before and after coming into contact with your pet(s)
- arrange for someone else to help care for your pet(s) (e.g. dog walking)

(Options: Yes, No)

Q49 To what extent do you agree or disagree with the following statements? (Please tick one box for each statement) (Strongly agree to strongly disagree)
• If I had coronavirus I would be willing for the data from my mobile phone to be used by public health doctors to identify the places I had visited in the past 7 days
• There should be a law that allows public health doctors to access the mobile phone data of people with coronavirus to identify the places they had visited in the past 7 days
• During a national emergency we should be less concerned about the privacy of our data

How have events affected you?

The following questions all relate to how recent events might have affected you.

Q50 Thinking over the last two weeks, how often have you been bothered by the following problems? (Please select one option for each row) (Options: Not at all, Several days, More than half the days, Nearly every day)

- Feeling down, depressed, or hopeless
- Little interest or pleasure in doing things
- Feeling tired or having no energy
- Trouble falling or staying asleep, or sleeping too much
- Poor appetite or overeating
- Feeling bad about yourself – or that you are a failure or have let yourself or your family down
- Trouble concentrating on things, such as reading the newspaper or watching television
- Moving or speaking so slowly that other people have noticed? Or the opposite, being so fidgety or restless that you have been moving around a lot more than usual?
- Thoughts that you would be better off dead, or of hurting yourself in some way?

Q51 In the past month, thinking about a stressful event related to the coronavirus outbreak have you…(Yes, No)

- had nightmares associated with your involvement with the event(s) or thought about your involvement with the event(s) when you did not want
- tried hard not to think about your involvement with the event(s) or went out of your way to avoid situations that reminded you of your involvement with the event(s)?
- been constantly on guard, watchful, or easily startled?
- felt numb or detached from people, activities, or your surroundings?
- felt guilty or unable to stop blaming yourself or others for your involvement with the coronavirus outbreak or any problems your involvement with the event(s) may have caused?

Q52 Thinking over the last two weeks, how often have you been bothered by the following problems? (Please select one option for each row) (Options: Not at all, Several days, More than half the days, Nearly every day)

- Feeling nervous, anxious or on edge
- Not being able to stop or control worrying
- Worrying too much about different things
- Trouble relaxing
- Being so restless that it is hard to sit still
- Becoming easily annoyed or irritable
- Feeling afraid as if something awful might happen

Q53 Over the last two weeks, please indicate how angry you have been feeling about being told to self-isolate? (Please select the option that BEST applies to you)
Not at all
Somewhat
Moderately
Very much

About you

Q54 What is your age?
18 to 24
25 to 44
45 to 69
69+

Q55 Are you...
Male
Female
Other
Prefer not to say

Q56 To which of these groups do you consider you belong?
White-British
White-Irish
White-other
Chinese
Asian
Black or Black British
African
Mixed
Other
Would prefer not to say

Q57 Which of the following best describes your education level? (if you have more than one job, please select the option that best describes your main job, i.e. the job you do most often)
Degree or above
Below degree level
Other
None (no formal qualification)
Would prefer not to say

Q58 Which of the following best describes your current employment status?
Working full time (30 hours a week or more)
Usually working full time (30 hours a week or more), but currently on leave or furloughed
Working part-time (8-29 hours a week)
Usually working part-time (8-29 hours a week), but currently on leave or furloughed
Stay at home parent/homemaker/housewife or househusband
Unemployed (registered or in process of registering)
Unemployed (not registered but looking for work)
Retired
Student
Don't know
Would prefer not to say
Other

Q59 At the time you were contacted by Public Health England, did you have any long-term illness, health problem or disability?
No
Yes

Q60 Please use this space to provide any additional comments about your experience of self-isolation:
