# Supplementary material for: Understanding adherence to self-isolation in the first phase of the COVID-19 pandemic in England: a cross-sectional mixed-methods study
Source: BMC Public Health. 2023 Oct 23;23:2074. doi: 10.1186/s12889-023-16674-9 (PMC10594856; doi:10.1186/s12889-023-16674-9)
Supplement: Supplementary file 3 — Additional file 3. [file 12889_2023_16674_MOESM3_ESM.pdf]

Self-isolation interview guide: Contact tracing group (Group 1)

(People contacted by Public Health England through contact tracing activities)

Introduction

Thank you for agreeing to take part in this interview. Before we start, I'd just like to remind you that all information that you give will be confidential, and any published data from these interviews will be anonymous. I'd also like to remind you that I am recording this interview with a digital recorder – this recording will only be used to allow us to analyse the data collected and audio recordings will be used for research purposes only.

We are interviewing people who were advised to self-isolate by Public Health England during the containment phase of the outbreak response. Public Health England were identifying and contacting people who had come into contact with a confirmed case were advising them to self-isolate, this is known as contact tracing. We are trying to find out what it was like trying to self-isolate, especially any difficulties you encountered, so we can provide advice and support for other people who have to self-isolate.

Experiences of contact tracing

Can you start off by telling me about the first time Public Health England informed you that you had come into close contact with a confirmed case of COVID-19/had tested positive for COVID-19?

Prompts

- *Did you know you know you had come into contact with a case before being contacted by Public Health England?*
- *Was the initial contact made by phone or text message?*

How did you feel about being identified as a contact?

Prompts

- *Was there anything you were particularly concerned about?*
- *Did contact with Public Health England change how you felt?*
- *Did anyone react differently to you since you were identified as a contact?*

Or

How did you feel about being identified as a case?

Prompts

- *Was there anything you were particularly concerned about?*
- *Did contact with Public Health England change how you felt?*
- *Did anyone react differently to you since you were identified as a case?*

Can you talk me through what you remember being said when you were contacted by PHE?

Prompts

- *Were you asked to self-isolate?*
- *Were you asked about your symptoms?*
- *Were you asked to monitor your own health?*
- *Were you asked to avoid contact with other people in your home?*
- *Was there anything you found confusing or didn't fully understand?*
- *How did you feel about the advice you received?*
- *If you were not asked to self-isolate, did you subsequently develop symptoms?*

Information and advice

Did you get any information or advice from other places?

Prompts

- *Internet*
- *Media*
- *Social media*
- *Friends/family*

What did you think of this information or advice?

Prompts

- *Did any of it conflict with PHE guidance?*
- *What sources did you find most reliable?*

[For those who were asked to or decided to self-isolate]

Experiences of self-isolation

You were asked to self-isolate. What does that mean in your view?

Thinking back to that time, how did you feel about being asked to self-isolate?

- *Would you feel differently if you were asked to self-isolate now?*

Can you tell me about your experiences of trying to self-isolate?

Prompts

- *Can you tell me what steps you took at first?*
- *Did anything change over time?*
- *Main problems with trying to self-isolate?*
- *Day to day living arrangements*
- *Family or other household members*

- *Work/study*
- *Finances*
- *Solutions to help with these problems?*

[Only for people who do not live alone]

- *Can you tell me about anything you did to self-isolate from other people in your household?*
- *How important did you think it was to self-isolate from other people in your household?*
- *What difficulties did you have trying to self-isolate from other people in your household?*
- *What might have helped you self-isolate from other people in your household?*

Isolating away from home

In some countries, accommodation is offered to help people with coronavirus or people who are at higher risk if they catch coronavirus self-isolate outside their own home. This is done so that coronavirus does not spread to other household members, especially if they are at higher risk.

How would you have felt about being offered accommodation for you to self-isolate away from your home while you were ill?

Prompts

- *What might have stopped you from taking up this offer?*
- *What might have encouraged you to take up this offer?*

[If they have vulnerable family members]

How would you have felt about accommodation being offered for any household members at higher risk for coronavirus to self-isolate away from the home while you were ill?

Prompts

- *What do you think might have stopped them from taking up this offer?*
- *What might have encouraged them to take up this offer?*

We realise that it may not be possible for everyone to follow all the advice to self-isolate – can you tell me about the times when it was not possible for you to follow the advice that you had received?

Prompts

- *Food supplies & medicine*
- *Work/livelihood*
- *Caring responsibilities*
- *Caring for animals*
- *Outdoor space*

Do you feel that self-isolation had an impact on your health and wellbeing in any way?

Prompts

- *Were there any aspects that particularly affected you?*
- *Was there anything that affected your physical health?*
- *Was there anything that affected your mental health?*

Support

What forms of support did you find you needed during self-isolation?

Prompts

- *Practical support*
- *Social support*

What forms of support were you able to get and who from?

Prompts

- *Public Health England*
- *Other health services*
- *Your employer/school/university*
- *Friends/family*
- *Neighbours*
- *Voluntary organisations*
- *Do you think there's any other support that might have been helpful?*

Information and advice

Did you get any information or advice from other places?

Prompts

- *Internet*
- *Media*
- *Social media*
- *Friends/family*

What did you think of this information or advice?

Prompts

- *What sources did you find most reliable?*

Thinking about the lockdown, how was your experience of self-isolation different?

Open comments [everyone]

Overall, what do you think of the advice about self-isolating?

What would you want to tell someone who has just been asked or decided to self-isolate?

Do you think self-isolating now would be different from when you were asked to do it?

Is there anything else you would like us to know about your experience that we haven't already covered?

Additional questions – do not ask if long interview

When the government announced lockdown, how clear did you find the advice you were given?

- *How does it compare with the advice you were given when you were contacted by Public Health England?*

If a COVID vaccination was available for free on the NHS, how would you feel about it?

- *Would you have it?*
- *Why/why not?*
